# Supplementary material for: Patterns of Intron Gain and Loss in Fungi
Source: PLoS Biol. 2004 Nov 30;2(12):e422. doi: 10.1371/journal.pbio.0020422 (PMC532390; doi:10.1371/journal.pbio.0020422)
Supplement: Table S1 — Also available at http://genes.mit.edu/NielsenEtAl/. (4.3 MB ZIP). [file pbio.0020422.st001.zip › NielsenEtAl/html/863.html]

AN3830.1.NCU02450.1.MG07224.1.FG00296.1


```
 CLUSTAL W (1.82) Multiple Sequence Alignments - Introns Inserted


Sequence 1: MG07224.1	618 aa
Sequence 2: FG00296.1	581 aa
Sequence 3: NCU02450.1	590 aa
Sequence 4: AN3830.1	587 aa
Alignment Length: 646 aa
Number Identitical Residues: 298 aa
Alignment Score (without introns) 14588


MG07224.1 	MAAPSDLEVTATKINGSSCQETVTNGDGINGPNGCSVAPATPATPTTPVMTVTRPSRTPS
NCU02450.1	--MASTLSAQPLLVNG-----------ASLQVVNGLNSE-RPASPS-------------S
FG00296.1 	--MPSADPIVAVPANG-----------AVNGTANGTNDDSRPQTP---------------
AN3830.1  	-----------------------------MTENASMNGAATPLSSR--------------
          	                                       : * :.               

MG07224.1 	LTGLSLTEYSANPSPPAESKRR-----KAKKVIPDDYILPSGHPD~--------------
NCU02450.1	VGNLALTEYSAMPSPPSEDATAN----KMKRIVPDEFLLPNGHPD0--------------
FG00296.1 	MTGMALTEYSANPSTPSAEKQA-----RLKEIVPEEYLLPTGYPD0--------------
AN3830.1  	LSNLALTEYSAIPTPTSEKEEYKGPDSPPAWDIPDAFLLPNGYPD~VRHVPAAYPNRSTA
          	: .::****** *:..: .   ....:     :*: ::**.*:**     .:: .. :::

MG07224.1 	------YLRLIL--TSKVYEVCKETSLTHAVNLSNRLECKVLLKREDEQPVFSFKLRGAY
NCU02450.1	------YLRLIITSYPRVREVCSEVPLTRAVNLSNRLECNVLLKREDEQPVFSFKLRGAY
FG00296.1 	------YLRLIAGATSRVYEACKVTPLTHAINLSNRLECNVLLKREDEQPVFSFKLRGAY
AN3830.1  	NNRYLQYLKLIL--TSRVYEITTESPLHHAVNLSNRMECRVLLKREDLLPVFSFKLRGAY
          	..   .**:**    .:* *  .  .* :*:*****:**.*******  ***********

MG07224.1 	NKMAHLDRAQSWRGVVCCSAG~NHAQGVAFSARKLKIPATIVMPEGTPSIKHLAVARMGG
NCU02450.1	NKMAHLDPKESWKGVVCCSAG~NHAQGVAYSARKLKIPATVIMPKGTPSIKHLNVSRMGS
FG00296.1 	NKMAHLDPKKSWKGVVCCSAG~NHAQGVAYSARKLKIPATIVMPEATPSIKHLNVARLGG
AN3830.1  	NKMAHLTDEQRWKGVIACSAG1NHAQGVAYSARKLKIPATIVMPSGTPAIKHLNVARLGG
          	******   : *:**:.**** *******:**********::**..**:**** *:*:*.

MG07224.1 	HVVLHGPDFDAAKEECARREKHDGLINIPPFDDPYVIAGQGTIGMELLRQTNLQKLEAIF
NCU02450.1	HVVLHGADFDEAKEECARRAKLDGLINIPPFDDPYVIAGQGTIGMEILSQTNLQKLQAIF
FG00296.1 	HVVLHGADFDAAKEECARREVQDGLINIPPFDDPYVIAGQGTIGNELFGQVNMAKVEAIF
AN3830.1  	SVVLHGNDFDAAKEEAHRREKQHGLTSIPPFDDPYVIAGQGTIGMEILRQANLDKLEAVF
          	 ***** *** ****. **   .** .***************** *:: *.*: *::*:*

MG07224.1 	CCVGGGGLIAGIGVYVKRIAPHIKIIGVETHDANAMTQSLAKGERVLLKDVGLFADGAAV
NCU02450.1	CAVGGGGLIAGVGVYVKRIAPHVKIIGVETYDADAMTQSLAKGERVLLQDVGLFADGAAV
FG00296.1 	CCVGGGGLIAGIGLYVKRMAPHVKIIGVEAQDANAMAQSLKKGERVLLKEVGLFADGAAV
AN3830.1  	CAVGGGGLISGVGVYIKRIAPHVKVIGVETHDANAMAQSLDEGSRVLLNEVGLFADGAAV
          	*.*******:*:*:*:**:***:*:****: **:**:*** :*.****::**********

MG07224.1 	RTVGEENFRICQEVIDEMVQVTTDEACAAIKDIFEDTRSVVEPAGALALAGLKKWVAANP
NCU02450.1	KTVGEETFRICQEVVDEMVQVTTDEACAAIRDMFEDTRSIIEPAGALAVAGLKKWVAANP
FG00296.1 	KIPGEETFRICKEVIDDVIEVSTDEICAAIKDMYDDTRSGLEPAGALSIAGLKKYVSQNP
AN3830.1  	KSVGSECWRVAREVVDEIILVSTDETCAAIKDAFEDTRSIIEPAGALAIAGLKKYVAKRP
          	:  *.* :*:.:**:*::: *:*** ****:* ::**** :******::*****:*: .*

MG07224.1 	STDSSRSLVAITSGANMNFDRLRFVAERANVGEGREALLAARIPEKPGSFAKLIEAIMPN
NCU02450.1	SADPTRSVVAITSGANMDFDRLGFVSARAKYGEGKEALLSVTIPERPGAFAELVNAIMPH
FG00296.1 	SDDSKRNLIAVTSGANMNFDRLRFVAERATMGEGKEALLSVQIPERPGAFSELINNIMPH
AN3830.1  	SPNTSRELVAITSGANMDFDRLRFVAERAALGEKKEALLSVKIPEQPGAFAKLVEVVLPH
          	* :..*.::*:******:**** **: **  ** :****:. ***:**:*::*:: ::*:

MG07224.1 	NVTEFTYRYTDGEVANIMLGLSLTAPVAQRAHQLQLLMGRIAEAG-MEVTDLSGDELAKS
NCU02450.1	AITEFSYRYATESKANVMVGISLADSGSRRAEQLQTIIGRIKQSGDMDVTDLSADELAKS
FG00296.1 	GVTEFSYRYSTDEVANILIGVSLTAPAHQRSEELRSLIDRIQSNN-MTVTDLSNDELAKS
AN3830.1  	AVTAFNYRYADETTADVLMGISLSASTGQ--EDLAKIMTELTRNG-MDSKDLSDDELAKR
          	 :* *.***:    *::::*:**: .  :  .:*  :: .:   . *  .*** ***** 

MG07224.1 	HVRYLVGGRAKVPNERLYMFEFPERPGALEKFLATLRPRYNISLFQYRNYGSDVGKILAG
NCU02450.1	HLRYMVGGRSNVPKERLYTFRFPERPGALERFLRLLRPKYNISLFQYRNYGGDIGQVLTG
FG00296.1 	HIRYLVGGRSGVPNERLYMFTFPERPGALEKFLVTLRPKFNISLFQYRNYGGDVGKIVTG
AN3830.1  	HLRFLVGGRSEVKDERLFMFEFPERPGALAKFLRTLRPSQNISLFHYRNYGGDVGKVLAG
          	*:*::****: * .***: * ******** :**  ***  *****:*****.*:*::::*

MG07224.1 	IQCPDGEMPELVKFLKEIGYPWFDC~TQSEVAKTFLGGPGVNEE~GAVPL
NCU02450.1	IQCPDEEVGELQSFFKEIGYPWEDS1DPHYKYVGLPSSNFCERR2-----
FG00296.1 	ILCPDEEVTELHNFLRKIGYPYEDC~TESPVFKTFLRS------~-----
AN3830.1  	IQCPECEKEQLEAFLKDLGYPFTEE~TQSETYKTFLRR------~-----
          	* **: *  :*  *::.:***: :          :
```
